# MCA-Mamba-2: A Multi-Channel Attention State-Space Network for Ultrasonic Guided Wave Damage Detection in Small-Diameter Pipes

**DOI:** 10.3390/s26185836

**Published:** 2026-09-15

**Authors:** Qiuyu Yu, Ruohua Zhou, Zhengxian Liang, Yajun Liu

**Affiliations:** School of Electrical and Information Engineering, Beijing University of Civil Engineering and Architecture, Beijing 102616, China; 2108110020002@stu.bucea.edu.cn (Q.Y.); 1108140024012@stu.bucea.edu.cn (Z.L.); lyj2100@hebiace.edu.cn (Y.L.)

**Keywords:** damage detection, ultrasonic guided waves, Mamba-2, state-space model, small-diameter pipe, structural health monitoring

## Abstract

Ultrasonic guided wave (UGW) inspection of small-diameter pipes is complicated by the narrow usable frequency band and by strong susceptibility to measurement noise, both of which degrade the reliability of damage identification. This study proposes a multi-channel attention Mamba-2 (MCA-Mamba-2) network that couples a convolutional front end with a bidirectional Mamba-2 state-space backbone and combines multi-head self-attention with channel attention for feature fusion. The network takes as input the four waveforms recorded simultaneously by a circumferential receiver array so that the input tensor has four physical channels that are subsequently expanded into learned feature channels. A dataset of ten damage states, spanning non-through-wall abrasions, through-wall holes and one multi-defect case, was acquired on a 20 mm outer-diameter steel pipe specimen. Averaged over three independent runs, MCA-Mamba-2 reaches 98.01 ± 0.85% accuracy on noise-free signals and 81.09 ± 1.74%, 83.65 ± 1.34%, 82.49 ± 0.20% and 84.15 ± 1.04% on 0 dB test sets contaminated with white, pink, Gaussian and Laplacian noise respectively, exceeding the Mamba-2 baseline in all sixteen tested noise-type and SNR combinations, by up to 17.87 percentage points. An ablation study attributes the improvement principally to the convolutional mixing block. A cross-noise evaluation reveals that accuracy drops by 59.80 percentage points, on average, at 0 dB when the training and testing noise distributions differ, indicating that the matched-noise accuracies are not indicative of robustness to unknown noise conditions; however, a single model trained on a mixture of all noise types and SNR levels recovers an average accuracy of 91.21% across the sixteen test conditions. Because each damage state was recorded in a single acquisition session, the dataset was partitioned at the level of individual records rather than by session, and the reported accuracies should be interpreted as an upper bound on the performance attainable on independently acquired data. The results were obtained on a single pipe geometry with fixed defect positions and synthetically added noise; extension to other geometries and to field measurements remains to be demonstrated.

## 1. Introduction

Small-diameter pipe structures are widely used in urban building applications such as heating, water supply, and gas supply. Ensuring their structural integrity and safe operation during service is paramount [1,2,3,4]. However, in-service conditions frequently expose these structures to diverse factors, including temperature fluctuations, mechanical loads, and chemical corrosion. Such conditions often lead to wall damage and rupture, resulting in leakage of the conveyed medium. This triggers corresponding economic losses and poses threats to personal safety [5,6,7,8]. Therefore, routine inspection and condition assessment of in-service pipes are essential to identify and repair damage before more severe consequences occur. Among various existing inspection methods, ultrasonic guided wave (UGW) testing technology stands out remarkably. In recent years, this technology has been widely applied in the field of Structural Health Monitoring (SHM) due to its unique advantages, demonstrating excellent performance and promising broad application prospects [9,10].

Guided ultrasonic wave technology is a non-destructive testing (NDT) method that enables inspection of target objects without causing collateral damage or impact. To ensure effective detection around the entire circumference of the pipe, multiple angularly symmetric transducers need to be uniformly installed along the circumference at a certain cross-section of the pipe. The guided waves emitted by these transducers interact with defects encountered during propagation, resulting in detectable changes in the wave signals. By analyzing the received signals, the presence of defects can be identified, and information regarding defect size, quantity, and severity can be further calculated [11,12]. Guided ultrasonic waves exhibit low energy attenuation and propagate over long distances, making them suitable for inspecting buried pipelines or areas obstructed by walls where visual or physical access is limited [13,14]. Additionally, this technology provides comprehensive coverage of a pipe’s entire cross-section and surface, ensuring detection of both circumferential and axial damage without omission [15]. Characterized by dispersion and multi-modal propagation [16], guided ultrasonic waves show varying sensitivity to different types and severities of defects. Whereas this enables detection of diverse pipe anomalies in a single inspection, it significantly increases the complexity of signal analysis. Traditional guided wave analysis predominantly relies on modal analysis and finite element methods (FEMs) for damage detection and characterization [17,18]. Building upon these foundational approaches, several researchers have advanced the methodology significantly. Yeung and Ng [19] proposed a finite element method capable of coupling torsional and flexural modes and analyzed the mode conversion phenomenon of guided waves in pipes by establishing crack elements. Bao et al. [20] employed FEM to analyze ultrasonic guided wave propagation in pipes with variable cross-sections. More recently, Qadri et al. [21] proposed an integrated computational framework combining analytical dispersion curves with finite element modeling, thereby improving computational efficiency. However, these approaches typically necessitate either the selective excitation of individual modes using specialized transducers or the implementation of modal decomposition techniques for multi-modal signals. The extracted modal data is subsequently analyzed through wave-defect interaction mechanisms to identify and characterize pipe defects.

In recent years, machine learning techniques have demonstrated outstanding performance in guided wave data analysis and defect detection for pipes, showing broad application prospects [22,23]. Lu et al. [24] proposed an ensemble data-driven model combining relevance vector machines with a multi-objective salp swarm algorithm optimization to accurately predict pipeline burst pressure across various corrosion conditions. Paialunga and Corcoran [25] developed an adaptive threshold method using Gaussian process regression that characterizes amplitude–temperature dependence at each A-scan location, outperforming optimal baseline subtraction for guided wave damage detection. Nerlikar et al. [26] extracted feature data from raw ultrasonic guided wave signals to train classification algorithms and validated the generalization capability of multi-class machine learning algorithms when structural material properties change. Ramatlo et al. [27] addressed the challenge of insufficient experimental data in ultrasonic guided wave inspection by generating synthetic rail-defect feature data using Variational Auto-Encoders (VAEs). However, machine learning methods require manual effort for complex data exploration and feature selection, with selection criteria often influenced by subjectivity and experiential bias. In contrast, deep learning approaches have relatively overcome these limitations while achieving superior accuracy on identical tasks.

In ultrasonic pipeline damage detection, research using deep learning methods focuses on achieving three key tasks: defect type identification, damage severity assessment, and location localization [28,29]. Zhang et al. [30] applied convolutional neural network (CNN)-based deep learning to ultrasonic guided wave signal processing for pipeline damage detection, demonstrating effectiveness and robustness under noise interference and temperature variations. Shang et al. [31] developed a hybrid model combining CNN and long short-term memory (LSTM) with twenty-nine predetermined features for ultrasonic guided wave analysis, achieving high accuracy in damage classification. Zhang et al. [32] investigated the identification of damage types, locations, and severity levels under various noise environments using a convolutional neural network. To enhance the interpretability of deep neural networks, Tang et al. [33] leveraged the LIME (Local Interpretable Model-agnostic Explanations) method to mine the correlations between output features and recognition results, proposing an MS-1DCNN-based ultrasonic guided wave pipeline structural health monitoring (SHM) model to achieve accurate identification of crack propagation levels in pipelines. Ai et al. [34] transformed ultrasonic guided wave signals received by multiple sensors into superimposed spectrograms and achieved precise localization of pipeline cracks in different orientations, lengths, and regions using the SEResNet50 deep neural network.

The aforementioned methods demonstrate that deep neural networks possess the capability to extract key features from ultrasonic guided wave signals and identify pipeline defects. However, these approaches are predominantly implemented on pipelines exceeding 50 mm in diameter, which are commonly used in oil and gas transmission, chemical industrial production, and major water supply pipelines [35,36]. In contrast, pipes in urban environments typically have smaller diameters. The reduction in diameter causes defects of practical interest to become small relative to the guided-wave wavelength. For the 20 mm outer-diameter specimen studied here, the L(0,2) wavelength at 80 kHz (λ≈58.9 mm) was derived from the phase velocity, which was computed in MATLAB R2020b (The MathWorks, Inc., Natick, MA, USA)using the PCDISP toolbox [37]. This toolbox solves the exact Pochhammer–Chree (Gazis) eigenvalue problem [38] for a homogeneous, isotropic, linearly elastic, hollow cylinder with traction-free inner and outer surfaces in vacuo. Thus, the circumferential widths of 3–9 mm considered here correspond to w/λ=0.051–0.153, placing the scattering firmly in the Rayleigh regime [39,40]. In this regime, the reflected energy decreases steeply with w/λ, rendering the defect echo weak relative to the direct arrival. This motivates the use of a network capable of preserving low-amplitude, temporally localised detail and is the reason why methods validated on larger pipes cannot be assumed to transfer directly [41].

Mamba-based architectures have been applied to a range of one-dimensional signal analysis tasks, including EEG decoding [42,43], multi-lead ECG classification [44], and—closest to the present setting—guided wave damage detection in composite plates [45]. In those settings, the discriminative information is quasi-stationary and distributed broadly along the sequence, so a state-space backbone preceded by a generic convolutional tokenizer can be applied to the raw record with little further modification. UGW records acquired in pulse-echo configuration on small-diameter pipes violate this assumption in two ways, and each dictates a specific design choice. First, the informative content is concentrated in a few wave packets whose arrival times are fixed by the pipe geometry and the group velocity of the excited mode. The convolutional front end of the MambaConvBlock is therefore not a generic tokenizer: its receptive field is matched to the duration of a single packet so that each packet is resolved locally before the state-space layer models the time-of-flight relations between them. Second, the record is dominated by components that carry no damage information—the excitation leakage at the transducer and the reflection from the far pipe end—while the defect signature appears as a comparatively weak perturbation superimposed on this background. Representations that pool aggressively over time are governed by the dominant arrivals, so an attention stage is used to re-weight the weaker components before global pooling. The multi-receiver input follows from the same reasoning: unlike time-aligned ECG leads [44], receivers distributed around the circumference at the excitation end respond differently to non-axisymmetric scattering, so the channel dimension carries information about the circumferential extent and position of the damage that no single channel resolves. Thus, the contribution of this work is not the use of Mamba-2 as such but the coupling of these choices and their systematic evaluation for damage-severity classification in small-diameter pipes; their individual contributions are isolated in Section 3.2.4.

To address the above challenges, this paper proposes a novel damage-state analysis method for ultrasonic guided wave-based structural health monitoring (SHM) of small-diameter pipes. The method develops a one-dimensional multi-scale Mamba model, whose core innovation lies in integrating the Mamba-2 state-space model with convolutional neural networks through a hybrid module, and incorporating an attention mechanism to achieve efficient fusion of multi-dimensional features. Its bidirectional processing strategy captures temporal dependencies, thereby enhancing the extraction of critical damage features. The study focuses on pipe structures with an outer diameter of only 20 mm, investigates the correspondence between extracted features and damage categories, and comprehensively validates the reliability of the proposed approach through comparative analysis with existing deep learning techniques. The method achieves superior accuracy over other models on both clean signals and noisy signals under various signal-to-noise ratios, including white noise, pink noise, Gaussian noise, and Laplacian noise.

The remainder of this paper is structured as follows. Section 2 introduces the theoretical principles of dispersion curves for ultrasonic guided waves and describes the deep learning model architecture employed in this study. Section 3 details the signal acquisition scheme for pipe damage evaluation and presents the physical experiments validating the effectiveness of the proposed method. Finally, Section 4 provides concluding remarks.

## 2. Theoretical Background and Model Architecture

The selection of the transmitter excitation signal is a crucial factor in ensuring the effectiveness of damage detection. Guided waves propagating longitudinally in hollow cylinders can be classified into three distinct modes based on previous research [46]: longitudinal (L(0,n)), torsional (T(0,n)), and flexural (F(m,n)), where the m and n indices correspond to the circumferential order and mode number, respectively. The flexural mode is non-axisymmetric, whereas the torsional mode involves only circumferential displacement, analogous to the shear wave mode in plates. The longitudinal mode comprises axial and radial displacement, exhibiting characteristics similar to Lamb waves in plates. When ultrasonic guided waves propagate through pipes, their wave velocities vary with frequency, manifesting dispersion properties.

**Figure 1:**
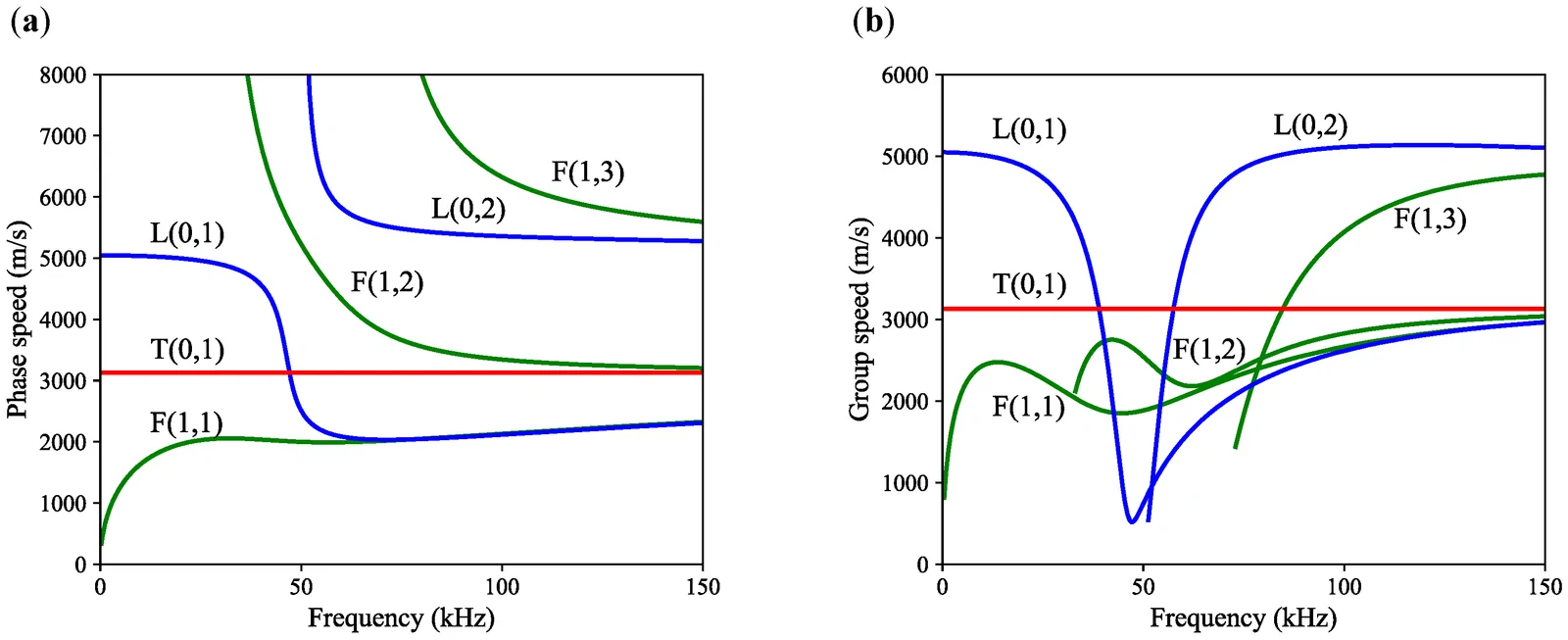
Dispersion curves of the test pipe. (**a**) Phase velocity; (**b**) group velocity.

The experiment employs a steel test pipe with an elastic modulus of 210 GPa, a Poisson ratio of 0.3, and a density of 7850 kg/m^3^. Its inner diameter is 14 mm, its outer diameter is 20 mm, and its length is 1.5 m. The dispersion curves shown in Figure 1 were computed by solving the Pochhammer–Chree (Gazis) eigenvalue problem [38] for a homogeneous, isotropic, linearly elastic hollow cylinder with traction-free inner and outer surfaces in vacuo using the PCDISP toolbox [37] in MATLAB R2020b; attenuation and loading by the surrounding medium were neglected. Substituting the Helmholtz potentials (ϕ and $\psi_{\theta }$), expressed through Bessel functions of arguments αr and βr with $\alpha^{2}=\omega^{2}/c_{L}^{2}-k^{2}$ and $\beta^{2}=\omega^{2}/c_{T}^{2}-k^{2}$, into the traction-free conditions ($\sigma_{rr}=\sigma_{rz}=0$) at r=a=7 mm and r=b=10 mm yields a 4×4 homogeneous system whose non-trivial solutions require detD(ω,k)=0 [38]. The real roots ($k_{m}\left(\omega \right)$) of this characteristic equation were tracked over 0–200 kHz [37], and the phase and group velocities were obtained as $c_{p}=\omega /k$ and $c_{g}=d\omega /dk$. The stated elastic constants correspond to bulk longitudinal and shear velocities of $c_{L}=6001$ m/s and $c_{T}=3208$ m/s. The excitation signal, characterized by a frequency of 80 kHz, is represented mathematically as:

(1) $$D\left(t\right)=A\left(1-\cos\frac{2\pi f_{c}t}{n}\right)\sin\left(2\pi f_{c}t\right).$$

where *A* is the peak amplitude of the excitation waveform. The waveform was generated in normalised form with A=0.5 (dimensionless). The carrier frequency is $f_{c}=80$ kHz. The n=5 parameter is the number of carrier cycles contained in the Hann-windowed tone burst: the $1-\cos(2\pi f_{c}t/n)$ envelope completes exactly one period over $0\leq t\leq n/f_{c}$, giving a burst duration of $n/f_{c}=62.5$ µs and a −6 dB bandwidth of approximately 30 kHz centred on $f_{c}$. A narrow bandwidth is desirable because it limits the excitation of neighbouring modes; n=5 was selected as a compromise between spectral purity and temporal resolution.

### MCA-Mamba-2 Network

To efficiently process long-sequence models, Gu and Dao proposed Mamba, an architecture built on selective state-space models [47]. The Mamba model integrates state-space theory, hardware-aware algorithms, and dynamic parametrization mechanisms, significantly enhancing modelling efficiency and computational efficiency in high-dimensional temporal data. Subsequently, Mamba-2 was introduced by Dao and Gu as an enhanced version of this architecture. Leveraging a novel structured state-space duality (SSD) algorithm, Mamba-2 achieves faster training speeds and improved capabilities in capturing long-range dependencies [48]. The method presented in this paper builds upon and extends the Mamba-2 model. Its applications span multiple domains, including time-series analysis [49,50], image processing [51,52], and remote sensing analysis [53].

The network input is a tensor of shape (B,4,L), where *B* is the batch size, the four channels are the waveforms recorded simultaneously by the four circumferentially distributed receivers (R1–R4), and *L* is the number of time samples. Each channel is standardised independently using statistics computed on the training partition only. The first convolution expands these four physical channels to 64 feature channels, and the two MambaConvBlocks further expand them to 128 and 256. The channel-attention branch of the AttentionPoolingBlock operates on these learned feature channels rather than directly on the four receivers; the term “multi-channel” in MCA-Mamba-2 therefore denotes the complete path from the four physical receiver channels through the learned feature channels and not attention over the receiver array alone.

**Figure 2:**
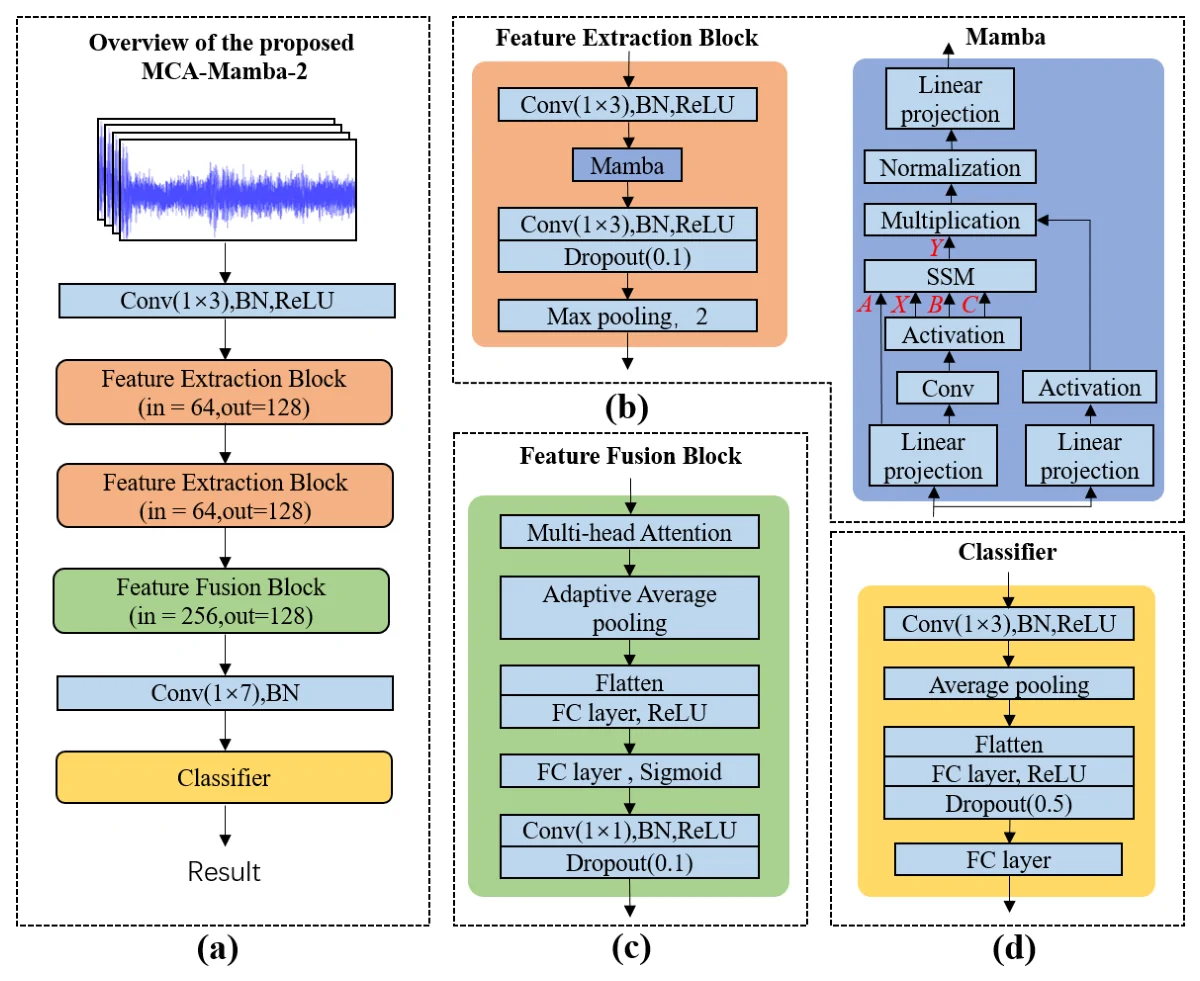
Architecture of the proposed multi-channel attention Mamba-2 (MCA-Mamba-2). (**a**) Overview of the proposed MCA-Mamba-2; (**b**) feature extraction block; (**c**) feature fusion block; (**d**) classifier. In (**b**), *A*, *B*, *C* and *X* denote the parameter matrices of the state-space model—the state transition, input, output and feed-through matrices, respectively—and *Y* denotes the output sequence of the selective scan.

Figure 2 illustrates the model’s complete structure, which adopts a design approach consisting of three stages. The initial phase focuses on extracting features at multiple scales, wherein the input signals, arranged as a four-channel tensor (B,4,L), undergo a one-dimensional convolution with kernel size of 7, stride of 1 and padding of 3 that expands the four receiver channels to 64 feature maps. This larger convolutional kernel captures richer local patterns, followed by batch normalization and a ReLU activation function for preprocessing. Subsequently, hierarchical feature learning is performed through two MambaConvBlocks with input–output channels of 64→128 and 128→256, respectively, progressively enhancing the abstraction level of features. Each MambaConvBlock additionally carries a residual connection from its input to its output, implemented as an identity mapping where the channel count is preserved and as a 1×1 convolution otherwise; since both blocks change the channel count (64→128 and 128→256), the 1×1 projection is used in both cases. The contribution of these connections is quantified in Section 3.2.4. Sequential downsampling is realized through stride-2 max pooling operations positioned after each MambaConvBlock, simultaneously alleviating computational demands and extending the scope of the receptive field. The second stage involves attention-based feature fusion, where the AttentionPoolingBlock reduces the feature dimension from 256 to 128 in the final layer, highlighting discriminative features through global information integration. At the third stage, the model handles classification in two steps: adaptive average pooling first normalizes variable-length features to uniform-length representations, which are then fed into a fully connected network with two layers for final classification. The two fully connected layers have input–output channels of 256→128 and 128→10, respectively, with a dropout probability of 0.5 inserted between them to enhance generalization capability.

The sequence modelling core of the model is implemented based on one-dimensional Mamba layers, which employ bidirectional processing strategies to enhance sequence understanding capability. The forward processing stream directly processes input sequences to capture temporal dependencies, whereas the backward processing stream reverses the sequence, processes it, then reverses it back to the original order. Finally, the feature representations from both directions are fused through addition operations. Both streams operate in a common latent width of 128 channels: a bias-free linear layer maps the incoming channels to this width before the two Mamba-2 scans, and a second bias-free linear layer maps the summed result to the number of output channels. To ensure computational efficiency, this layer pads the input sequence length to multiples of 64 and precisely crops it to the original sequence length during output. In the specific implementation, the Mamba layer adopts a relatively lightweight configuration: the state dimension (d_state) is set to 8, and the chunk size is set to 16, with an expansion factor of 2 and a head dimension of 64 (i.e., an inner width of 256 split into 4 heads), whereas the layer count is limited to 1 to minimize computational cost. This parameter configuration effectively controls model complexity while maintaining modelling capability.

For global integration of high-level features, an AttentionPoolingBlock module is incorporated, leveraging both multi-head self-attention and channel attention mechanisms. Long-range dependencies across various signal positions are captured through multi-head self-attention, where the head count equals the largest integer from dividing input channels by 64 (4 heads for the 256 input channels), while a 0.1 dropout rate is applied to improve generalization. The channel attention mechanism draws inspiration from SENet design philosophy, compressing features into global descriptors through adaptive average pooling, then learning of inter-channel correlations through a two-layer fully connected network (with the reduction ratio set to 4, i.e., 256→64→256) and, finally, mapping of weights to the range of [0, 1] through the sigmoid activation function. Each channel’s features are scaled according to their corresponding weights, thereby enhancing the model’s attention to key features. This dual attention mechanism simultaneously focuses on positional correlations within sequences and the importance of feature channels, aimed at enhancing the model’s global feature modelling capability. After self-attention and channel attention processing, the model reduces the dimensionality of these features through a 1×1 convolutional layer to ensure output feature dimensions meet subsequent classification requirements. The dimensionally reduced features are further processed through batch normalization, the ReLU activation function, and a dropout of 0.1 to maintain model stability and nonlinear representation capability.

The final stage of the model involves global feature extraction and classification. First, features are refined through a convolutional layer with a kernel size of 3 that maps the 128 fused feature channels back to 256, providing more precise input for the classification layer. Afterward, global average pooling compresses the features by converting each signal sequence into a fixed-size representation. A series of fully connected operations then follows: ReLU-activated feature refinement, dropout-based regularization to prevent overfitting, and linear transformation for classification output.

The model’s superior classification capability stems from its ability to extract latent features across multiple granularities in 1D signals, achieved by synergistically combining: (1) convolutional layers for local temporal features, (2) Mamba for temporal dependencies, (3) attention mechanisms (both self and channel) for holistic representation, and (4) pooling–convolution hybrids for hierarchical fusion. The model contains 1,182,586 trainable parameters, trained for 20 epochs using the Adam optimiser with a learning rate of 0.001, a batch size of 4 and gradient accumulation over 4 steps, giving an effective batch size of 16. Its combination of multi-level feature extraction, cross-layer aggregation, and noise incorporation enables the model to retain vital signal attributes while handling complex noisy scenarios, leading to enhanced robustness and modelling performance. The complete layer-by-layer configuration of the network is summarised in Table 1.

## 3. Experiments and Results

### 3.1. Experimental Setup

#### 3.1.1. Instrumentation and System Configuration

**Figure 3:**
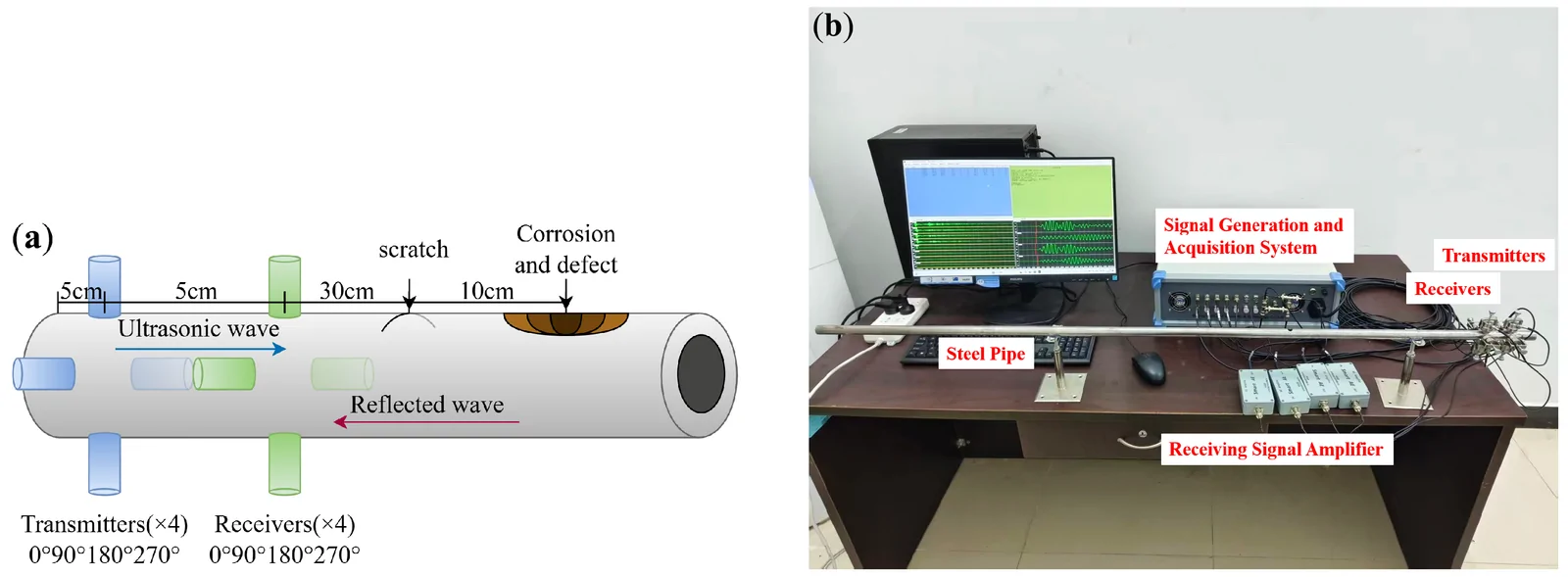
Experimental arrangement. (**a**) Schematic of a 1.5 m steel test pipe showing transmitters (T1–T4) at 5 cm and receivers (R1–R4) at 10 cm from the right end, the principal defect at 50 cm, and the shallow scratch at 40 cm (only in the “Hole 4 add crack” class); inset: circumferential positions of the transducer pairs and defects. (**b**) Photograph of the equipment.

**Figure 4:**
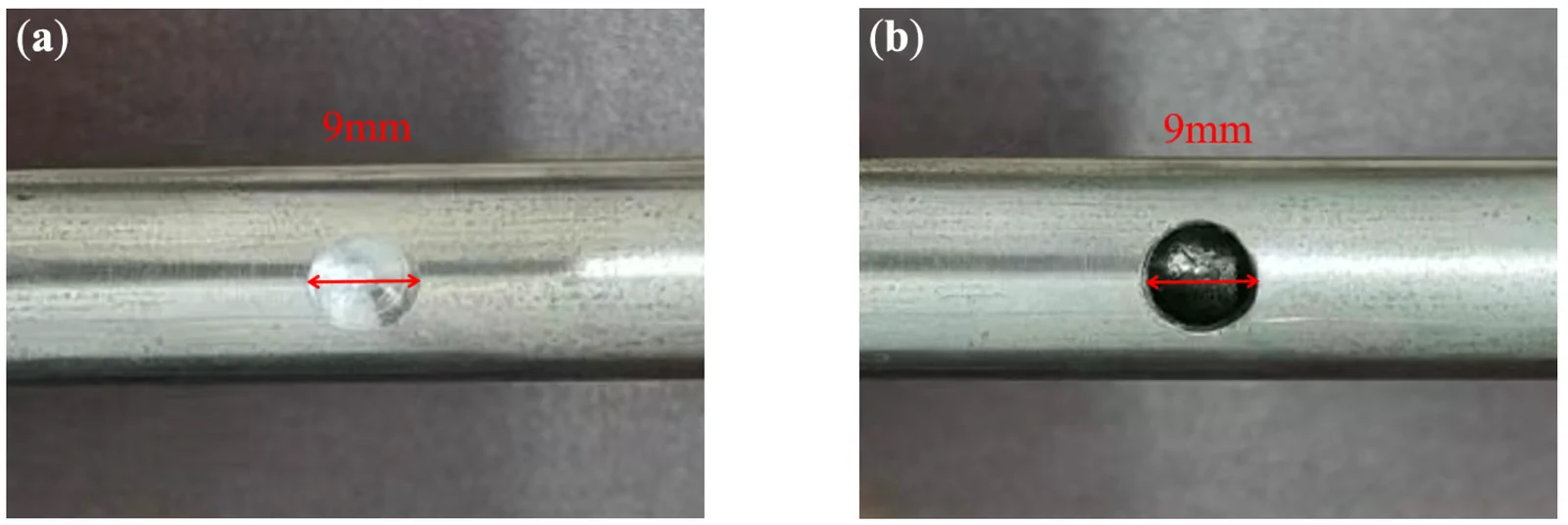
Differences between two damage types. (**a**) Abrasion; (**b**) hole.

The instruments used in the experiment are shown in Figure 3. Guided waves were excited and received using a piezoelectric transducer set, an arbitrary waveform generator and a data acquisition card (Beijing Softland Times Technology Co., Ltd., Beijing, China). The specimen was a seamless steel pipe machined by Dongguan Chang’an Xulin Metal Materials Trading Firm (Dongguan, China). Signal transmitters are installed at a position 5 cm from the right end of the test pipe, whereas signal receivers are installed at a position 10 cm from the right end. The sensors are arranged in arrays of four and positioned in parallel configuration, uniformly distributed at 0°, 90°, 180° and 270° around the circumference. Because the transmitter array lies closer to the right end than the receiver array, the wave packet generated at 5 cm first travels leftwards past the receivers at 10 cm, then propagates towards the defect region; the recorded signal therefore contains the direct arrival followed by the echoes reflected from the defects and from the far end. Damage is introduced at a location 50 cm from the rightmost end of the test pipe. For each experiment, the type and extent of damage are varied at the same location, with the differences detailed in Table 2. The geometrical distinction between the two damage types is illustrated in Figure 4. A shallow scratch, perpendicular to the axial orientation and located 40 cm from the right terminal at a circumferential angle of 0°, was introduced on the upper surface of the test pipe after the Hole 4 measurements had been completed in order to assess multi-damage detection performance. This scratch is therefore present only in the “Hole 4 add crack” class of Table 2 and absent from all other classes.

The ten classes span two factors. The first is the damage type: undamaged, a non-through-wall notch, a through-wall hole, and a multi-defect configuration. The second is the damage extent, parameterised here by the circumferential width and quantified by the resulting loss of cross-sectional area; within each type, the extent increases monotonically with the class index. The task addressed in this work is therefore a joint classification of damage type and extent over a discrete set of machined configurations rather than the regression of a continuous severity measure.

#### 3.1.2. Dataset Construction and Partitioning

For each damage state, the excitation was repeated under identical settings and every repetition was stored as one record. Each recording was acquired at a sampling rate of 3 MS/s; the waveforms from the four receivers in one recording were stacked to form a single sample of shape (L,4). The transducers were dismounted and re-coupled to the specimen before the measurements of each damage state so that every class was recorded under an independently established coupling condition; within a damage state, the repetitions share that single coupling condition. The samples are therefore repeated excitations of one physical configuration and are neither independent specimens nor segments extracted from a longer record. The per-class sample counts are listed in Table 2 and total 12,047 samples, which were partitioned into training, validation and test subsets in a ratio of 8:1:1, giving 9637/1205/1205 samples respectively.

The partition was performed at the level of individual records. Because all samples of a given class originate from the same pipe specimen, the same machined defect and a single coupling condition, records acquired in close succession are strongly correlated, and a record-level partition places correlated samples in both the training and the test subset. A group-wise partition at the level of acquisition sessions is not available for this dataset: each damage state was recorded in exactly one session, so assigning whole sessions to subsets would place every sample of a class on one side of the partition, and the classification task would degenerate. Resolving this requires several sessions per damage state, each with an independent re-coupling of the transducers, which was beyond the scope of the present acquisition campaign and is identified as future work in Section 4. The accuracies reported below should accordingly be read as an upper bound on the performance attainable on independently acquired data, and the point is restated among the limitations in Section 3.3.

All experiments were conducted on an NVIDIA Tesla V100S PCIe GPU (32 GB HBM2; NVIDIA Corporation, Santa Clara, CA, USA) with an Intel Xeon Gold 5218 processor (Intel Corporation, Santa Clara, CA, USA). The implementation is based on Python 3.10 and PyTorch 1.13.1 with CUDA 11.2. The selective state-space layer of Mamba-2 is our own pure-PyTorch reimplementation following the original paper; the fused CUDA kernels from the official mamba-ssm package were not used. To emulate an effective batch size of 16 within the available GPU memory, gradient accumulation over four steps was employed with a micro-batch size of four per step. All random number generators (Python 3.10, NumPy 1.23.5, PyTorch 1.13.1 and CUDA 11.2) were explicitly seeded, and torch.backends.cudnn.deterministic was set to True. Every configuration reported below—all methods under the noise-free condition and all methods under each of the four noise types and four SNR levels—was run three times independently with seeds {0, 1, 2}; the reported values are the means and corresponding sample standard deviations over these three repetitions.

#### 3.1.3. Noise Configuration

**Figure 5:**
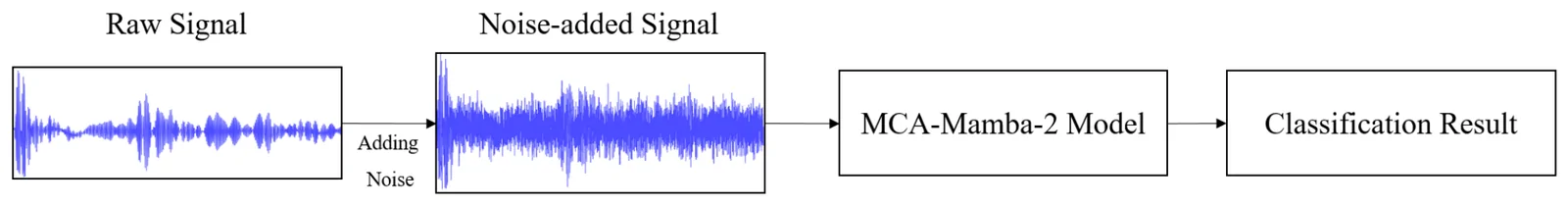
Deep learning-based ultrasonic guided wave pipe damage classification experimental procedure.

Figure 5 outlines the experimental methodology. The process begins with acquiring reference signals from the pristine test pipe. Controlled noise is then injected to emulate field conditions. Subsequently, the proposed technique is utilized to diagnose damage and quantify detection performance under these contaminated scenarios. Ultrasonic guided wave measurements were obtained from the test pipe in each of the ten damage states through sensor arrays, with acquisition conducted in interference-free environments to secure pristine signals. Given that real-world signal collection faces multiple noise perturbations, the addition of noise to the acquired data is required to validate the model’s classification resilience. The noise synthesis method used here follows the protocol described by Zhao et al. [54]. Noise is injected into all four receiver channels of every clean record, with each channel receiving an independent noise realisation. A single power normalisation is applied across the four channels so that the nominal SNR refers to the total power of the four-channel record rather than to any individual receiver. Consequently, channels with weaker responses are effectively noisier than those with stronger responses, which reflects the situation encountered in practice when a common acquisition setting is applied to the whole array. These noisy signals are then used to train the model in a supervised learning manner.

The four noise types are chosen so that the effect of the spectral shape can be separated from that of the amplitude distribution; Table 3 summarises the two properties for each type.

The power spectral density (PSD) of the incorporated, uniformly distributed white noise and pink noise is defined as follows:

(2) $$S\left(f\right)=S_{0}$$

(3) $$S\left(f\right)=\frac{K}{{\left|f\right|}^{\alpha }}$$

where frequency is denoted by *f* (Hz), $S_{0}$ is defined as the constant for power per unit frequency, and the spectral exponent (α) is set to 1 in the case of standard pink noise. To validate the model under different noise conditions, Gaussian and Laplace noise were added with the following probability density functions:

(4) $$p\left(x\right)=\frac{1}{\sqrt{2\pi \sigma^{2}}}e^{-\frac{{\left(x-\mu \right)}^{2}}{2\sigma^{2}}}$$

(5) $$p\left(x\right)=\frac{1}{2b}e^{-\frac{\left|x-\mu \right|}{b}}$$

where μ is defined as the mean that establishes the distribution’s centre, σ is given as the standard deviation indicating noise spread, and *b* is specified as the scale parameter associated with distribution breadth. In every case, the noise is first generated with the prescribed spectral shape and amplitude distribution and is then scaled to the power required by the target SNR so that the shape and the power are controlled independently.

Practical UGW inspection is affected by several noise contributors, including transducer self-noise, electronic instrumentation noise and ambient environmental interference. Because these contributions cannot be isolated in the laboratory, controlled synthetic noise is added to the clean signals so as to obtain repeatable and parametrically varied test conditions.

Two evaluation protocols are used and are reported separately. In the matched-noise protocol (Section 3.2.2), a separate model instance is trained from scratch for each noise type and SNR level and is evaluated on a held-out test set drawn from the same noise distribution. This protocol isolates the effect of signal quality on the classification task itself, but it does not measure robustness to noise conditions that were not seen during training. In the mismatched-noise protocol (Section 3.2.3), models are evaluated on noise types and SNR levels absent from their training data, and a single model trained on a mixture of all noise types and SNR levels is compared against the condition-specific models. It is the latter protocol that is relevant to field deployment, where the noise characteristics are not known in advance.

To comprehensively evaluate model robustness and classification efficacy across different signal quality scenarios, this investigation utilizes a dual-metric approach: classification accuracy and Signal-to-Noise Ratio (SNR). The latter, measured in decibels (dB), characterizes the strength ratio of the meaningful signal to the noise. A lower SNR value indicates poorer signal quality, which typically presents greater challenges for correct classification. The classification accuracy metric quantifies the fraction of test samples that are correctly categorized relative to the entire test population, with higher values indicating enhanced classification proficiency. Because the ten classes are of comparable size, the macro-averaged F1 score is additionally reported so that performance on individual damage states is not masked by the overall accuracy. Model performance under noise perturbation is evaluated by tracking accuracy variations across different SNR scenarios. The computational expressions for these metrics are presented as follows:

(6) $$\mathrm{SNR}\;\left(\mathrm{dB}\right)=10\log_{10}\left(\frac{\frac{1}{N}\sum_{i=1}^{N}x_{i}^{2}}{\frac{1}{N}\sum_{i=1}^{N}n_{i}^{2}}\right)$$

(7) $$\mathrm{Accuracy}=\frac{N_{\mathrm{correct}}}{N_{\mathrm{total}}}\times 100\%$$

where SNR is defined as the signal-to-noise ratio, $x_{i}$ is given as the amplitude corresponding to the *i*-th signal sample, $n_{i}$ is specified as the amplitude for the *i*-th noise sample, *N* denotes the total sample quantity, $N_{\mathrm{correct}}$ represents the quantity of accurate classifications in the test set, and $N_{\mathrm{total}}$ indicates the test set’s complete sample count.

### 3.2. Experimental Results and Analysis

#### 3.2.1. Classification on Noise-Free Signals

All baseline models were trained under an identical protocol: the same data partition (9637/1205/1205 signals for training, validation and testing), the same number of epochs (20), the same optimiser (Adam with a learning rate of $10^{-3}$, batch size of 4, and four gradient-accumulation steps, i.e., an effective batch size of 16), and the same model-selection criterion (the checkpoint with the highest validation accuracy). All models were trained from scratch with the same three random seeds (0–2) and evaluated on exactly the same test set, and Table 4 reports the mean and standard deviation over these three runs. Baseline hyperparameters were selected by a grid search over a hidden width of ∈{64, 128, 256} and depth of ∈{1, 2, 3}, using validation accuracy under the same selection protocol as the proposed model, with ties broken in favour of the smallest parameter count. The selected settings were a width of 64 and three layers for RNN, a width of 128 and one layer for LSTM, and a width of 64 and one layer for Transformer. Parameter counts are listed in Table 4 so that the comparison is not confounded by model capacity. The recurrent and attention baselines remain below 89% accuracy and show large seed-to-seed variation, whereas the plain Mamba-2 backbone reaches 92.15% with roughly half the spread. Since MCA-Mamba-2 has a larger parameter count than the plain baselines, a width-matched Mamba-2 baseline ($d_{\mathrm{model}}=384$, 1,123,902 parameters, i.e., 95% of those of MCA-Mamba-2) is reported in the penultimate row: scaling capacity alone lifts accuracy to 95.30%, while adding multi-scale convolutional attention gives a further 2.71 points (98.01% accuracy, 97.65% macro-F1) for only 5% more parameters, with the smallest variance of all six models. Whether this margin persists under noise is examined in Section 3.2.2.

#### 3.2.2. Classification Under Matched Noise

To comprehensively compare the damage recognition performance of the different models, all deep learning models were evaluated under four noise types (white, pink, Gaussian and Laplacian noise). For each noise type, a separate model instance was trained from scratch on noisy signals with signal-to-noise ratios (SNRs) of 0, 5, 10 and 15 dB, and each instance was evaluated on the test set sharing the same noise type and SNR. The results are summarised in Table 5.

All models degrade as the SNR decreases, but the extent of the degradation depends strongly on the noise type. Averaged over the five models, the accuracy loss between 15 dB and 0 dB is 17.61 percentage points for white noise, 24.46 percentage points for Gaussian noise, 27.94 percentage points for Laplacian noise and 31.14 percentage points for pink noise. Pink noise is therefore the most damaging of the four conditions. This is consistent with its 1/f power spectral density: although comparatively little pink-noise power falls within the excitation band around 80 kHz, the low-frequency content produces large-amplitude baseline fluctuations in the time domain, which dominate the waveform and mask the low-amplitude defect echoes. The effect is most pronounced for the RNN, whose accuracy under pink noise at 0 dB falls to 19.92%, which is roughly twice the 10% chance level of the ten-class problem and the lowest single entry in the table.

Despite this degradation, MCA-Mamba-2 attains the highest accuracy in all sixteen examined noise-type and SNR combinations, outperforming the RNN, LSTM, Transformer and Mamba-2 baselines throughout. Averaged over the four SNR levels, it exceeds the Mamba-2 baseline—the state-space model from which the proposed architecture is derived—by 5.91, 12.40, 7.49 and 4.27 percentage points under white, pink, Gaussian and Laplacian noise respectively. The largest single margin over Mamba-2 is 17.87 percentage points, attained under pink noise at 5 dB. Under the most demanding tested condition (0 dB), Mamba-2 is the strongest competing model under every noise type, and the corresponding margins over it are 4.24, 13.36, 5.31 and 9.21 percentage points. It should be noted that these results were obtained under the matched-noise protocol defined in Section 3.1.3, in which the training and test sets share the same noise type and intensity; they therefore characterise classification performance at a given signal quality rather than robustness to noise conditions not seen during training. The latter is examined separately in Section 3.2.3.

These results confirm the consistent advantage of the MCA-Mamba-2 model under different noise environments. The model captures temporal dependencies in signals through bidirectional sequence modelling and achieves multi-dimensional feature fusion by combining multi-head self-attention and channel attention mechanisms, effectively suppressing noise interference and focusing on critical damage features in the signals. The advantage is largest precisely where the baselines are weakest—under pink noise, where the margin over the best baseline reaches 17.87 points at 5 dB and 13.36 points at 0 dB—which is consistent with the multi-scale branches recovering echo structure that survives the low-frequency baseline drift.

Four entries in Table 5 do not increase monotonically with the SNR, and all of them belong to baseline models; MCA-Mamba-2 increases monotonically with the SNR under all four noise types. Two of the deviations are minor: between 10 and 15 dB, the Transformer loses 0.67 points under white noise, and between 0 and 5 dB, Mamba-2 loses 2.57 points under pink noise. In both cases, the two entries overlap within one run-to-run standard deviation, so the ordering of these pairs is not statistically resolved, and we do not read a systematic effect into it. Two deviations exceed that margin, both for Mamba-2 between 10 and 15 dB: 4.81 points under pink noise and 4.15 points under Gaussian noise. Because the noise realisations, the train/test partition and the initialisation are drawn independently at each SNR, adjacent columns are not nested samples of a single experiment, and a favourable draw at one SNR can outrank an unfavourable draw at the next. We report these entries as measured rather than smoothing them and note that the comparison of interest here is between models within a column, which is unaffected.

#### 3.2.3. Generalisation to Unseen Noise Conditions

Table 5 trains and tests each model under the same noise type and the same SNR and therefore measures matched-condition performance rather than robustness. We repeated the experiment with the two decoupled: keeping the MCA-Mamba-2 architecture fixed, four models were trained on a single noise type at 0 dB, and a fifth model was trained on a random per-sample mixture of all four noise types and all four SNR levels; each was evaluated on all 16 test conditions (four noise types × four SNR levels) over three seeds. Test noise is drawn from a generator seeded independently of the training condition, so every model in a column of Table 6 sees identical corrupted signals, and the comparisons are paired. The layout mirrors Table 5, with the model dimension replaced by the training condition, so its 0 dB column is the full cross-noise transfer matrix, and each row read horizontally is a cross-SNR curve.

The four underlined entries average 91.63%, whereas the twelve mismatched entries at 0 dB average 31.83%—a generalisation gap of 59.80 percentage points—so the accuracies of Table 5 are an upper bound rather than evidence of general robustness. What transfer survives follows the power spectrum, not the amplitude distribution: mismatched pairs sharing a spectral shape (white ↔ Laplacian, both flat; pink ↔ Gaussian, both $1/{\left|f\right|}^{\alpha }$) retain 52.44%, against 21.53% for pairs whose spectra differ—close to the 10% chance level of the ten-class problem. Transfer within a matched pair is nonetheless strongly asymmetric: pink → Gaussian retains 82.97%, while Gaussian → pink retains only 10.91%, so a shared spectral shape is necessary but not sufficient. Attenuating the test noise does not help either: the white- and Laplacian-trained models fall to 9.30–9.33% at every higher SNR, i.e., essentially every signal assigned to one class. Lowering the noise power is, itself, a covariate shift, and since the normalisation statistics are dominated by the training noise, a cleaner input falls outside the range the network was fitted on. The Gaussian-trained row needs a caveat: with a $1/{\left|f\right|}^{2}$ spectrum, nearly all noise power sits at very low frequencies, so at equal total power, little energy reaches the informative band, and the condition is effectively a mild one in disguise—which is also why that model transfers well to the other noise types at 15 dB (87.26–96.93%), where the test signal is likewise only lightly corrupted.

The last row of each group is one model, trained once on the mixture. Over the 16 conditions, it averages 91.21%, against 51.11% for the condition-specific models evaluated across the full SNR range of their own noise type. The cost is confined to the matched condition: at 0 dB, it averages 81.69% against 91.63%, corresponding to a loss of 9.94 pp, while elsewhere, it leads by up to 65.50 pp and is best in 7 of the 16 columns. All seven of these are at 5 dB or above, and none falls in the Gaussian test group, where the Gaussian-trained model remains best throughout for the reason just given. Thus, a single model exposed to diverse corruptions covers the whole operating envelope at a fraction of the cost of sixteen specialised ones and without requiring the noise condition to be known at inference time, and we adopt mixed-noise training as the recommended protocol. Two caveats remain: the four corruptions are synthetic, additive and stationary, so the gap above bounds robustness only within that family and not for the non-stationary, signal-correlated disturbances of a real acquisition chain, and the collapse at higher SNRs is partly an artefact of fitting the normalisation on noisy training data, which we report rather than remove because the same weakness is implicit in any single-condition protocol.

#### 3.2.4. Ablation Study

To quantify the contribution of each component of MCA-Mamba-2, we conduct a controlled ablation study. Starting from a Mamba-2 backbone with residual connections (A0), the convolutional mixing block, bidirectional state-space processing, multi-head self-attention (MHSA), and channel (squeeze-and-excitation, SE) attention are added cumulatively so that each configuration differs from the previous one by a single factor. Configurations A3 and A4 further isolate the two attention mechanisms by adding MHSA alone and channel attention alone on top of the same A2 base. The A0 backbone used here is wider than the plain Mamba-2 baseline of Table 4 and carries the residual connections of the proposed design, so its parameter count and accuracy are not directly comparable with that baseline. All other hyperparameters, the training schedule, and the data splits are held fixed. Each configuration is trained with three independent random seeds and evaluated under three conditions—noise-free, white noise at 0 dB, and pink noise at 0 dB. We report accuracy and, since the task has ten classes, the macro-averaged F1 score in Table 7, both as mean ± standard deviation, together with the parameter count of each configuration.

The full model attains the best value in every column of Table 7, and the components separate cumulatively along the chain of A0–A1–A2–A3–A5 under all three conditions. On clean signals, the backbone alone (A0) reaches 92.18%, and each addition improves on the previous configuration, with the convolutional mixing block contributing 2.18 points, bidirectional processing contributing a further 0.76, self-attention contributing 1.62 and the channel-attention branch contributing 1.27 so that A5 attains 98.01±0.85% accuracy and 97.65±1.02% macro-F1 with the smallest spread of the study. The same ordering is preserved under noise, where the chain rises from 76.52% to 81.09% under white noise and from 76.80% to 83.65% under pink noise, corresponding to gains of 4.57 and 6.85 points over the backbone. The single exception is A3 under white noise, which falls 0.75 points below A2; this is well within its own standard deviation of 5.24 points, and we do not read a systematic effect into it. Channel attention is effective only in combination: applied alone on the A2 base (A4), it lowers accuracy under both noise conditions, to 70.28±15.17% under white noise—a variance an order of magnitude larger than that of A2 itself—and to 75.60% under pink noise, whereas combining it with self-attention in A5 recovers 10.81 and 8.05 points respectively and returns the spread to 1.74 and 1.34 points. Macro-F1 tracks accuracy throughout, trailing it by 1.24 points for A5 under white noise and 1.25 under pink noise, so none of these differences is an artefact of class imbalance.

Taken together, the ablation shows that the components are complementary rather than redundant and that their effects are additive on clean signals and under both 0 dB corruptions alike. The margin of the full model widens as the signal quality falls—1.27 points over A3 on clean signals against 2.69 under white noise and 2.45 under pink noise—which is consistent with the attention stage re-weighting the weak defect echoes that the noise would otherwise mask. The parameter counts show that these gains are not simply bought with capacity: A3 uses 97% of the parameters of A5 yet trails it in all three conditions, and A4 uses more parameters than A2 while performing worse in all three. Because all results are averaged over three seeds and reported with their standard deviations, the ordering along the cumulative chain is well resolved on clean signals, where the spreads are around one point; under noise, the configurations that omit one of the two attention branches (A1; A3; and, in particular, A4) show spreads of 5 to 15 points, so the reliability of the full model—and not only its mean—distinguishes it from them.

#### 3.2.5. Sensitivity to the Training Configuration

Table 8 summarises the hyperparameter sensitivity and convergence behaviour of MCA-Mamba-2. Among the four examined learning rates, performance is stable across $10^{-4}$–$10^{-3}$: clean accuracy ranges from 96.42% to 98.01%, and 0 dB white-noise accuracy ranges from 76.85% to 81.09%, while only $5\times 10^{-3}$ causes a pronounced drop (82.47%/41.83%). The default of $\mathrm{lr}=10^{-3}$ achieves the highest accuracy under both conditions. Varying the batch size from 4 (effective batch size of 16) to 16 (effective batch size of 64) yields differences below 2.6 pp, confirming that the model is insensitive to this factor. Comparing the “Best at 20” column with the full 40-epoch accuracy shows that, for all configurations except $5\times 10^{-3}$, the gap is, at most, 0.22 pp, demonstrating that 20 training epochs are sufficient. These results confirm that the reported performance is not an artefact of a particular hyperparameter choice and that the training budget is adequate.

#### 3.2.6. Effect of the Training-Set Size

The comparisons reported so far use the full training partition and therefore establish that MCA-Mamba-2 outperforms the baselines without establishing when it does, which matters here because every recording corresponds to a physically machined defect state and the number that can realistically be acquired is a practical constraint. The comparison is restricted to Mamba-2 and MCA-Mamba-2, which differ only by the components introduced in this work, and is carried out under white noise at 0 dB, the condition under which the two are best separated; on noise-free signals, both approach the ceiling, and any margin defined there is bounded above by the residual error of the weaker model. The training pool was subsampled class-wise to 5%, 10%, 25%, 50% and 100% of its size, yielding 482 to 9637 recordings or roughly 48 to 964 per class. The subsets are nested, so successive points differ only by the recordings that were added rather than by the draw of a different sample, and the test partition is never subsampled, so the entries of Table 9 are directly comparable both with one another and with Table 5. To avoid confounding a reduction in data with a reduction in the number of gradient updates, the epoch budget was scaled inversely with the subsampling fraction and capped at four times the nominal value of 20, with the model retained being, in every case, the one with the highest validation accuracy. Each point was obtained from three independent runs with seeds 0–2, as elsewhere in the paper, and the reported values are the corresponding means.

Three observations follow. First, MCA-Mamba-2 leads at every one of the five sizes across a twentyfold range of data volume, so the ordering of the two models is not an artefact of the partition size used in the main experiments. Second, the margin does not close as data is added: in accuracy, it stays between 3.24 and 4.24 percentage points, and in macro-F1, it increases monotonically from 3.14 to 5.95. This is the opposite of what would be expected if the additional components merely compensated for a shortage of data, in which case the curves would converge once enough recordings were available. Third, and of most practical relevance, MCA-Mamba-2 trained on 2409 recordings reaches 77.85% accuracy, exceeding the 76.85% that Mamba-2 attains with the complete set of 9637; reaching a given accuracy under this condition therefore requires roughly four times fewer labelled recordings, which, for a dataset built from machined defect states, translates into a correspondingly smaller experimental campaign. The advantage is nonetheless smallest in absolute terms at the low-data end: at 482 recordings, both models fall below 70% accuracy and 63% macro-F1, so the proposed architecture reduces the error without making the ten-class task tractable at that scale. Thus, conditions under which MCA-Mamba-2 is worth its additional parameters are bounded on both sides, with the useful operating range lying between saturation on clean signals and unusable accuracy under severe data scarcity. The standard deviations contract monotonically as the training pool grows, from 4.82 to 2.95 points for Mamba-2 and from 3.15 to 1.74 for MCA-Mamba-2, with the proposed model being the more stable of the two at every size. For the two smallest pools, however, the margin between the models is of the same order as this spread, so the ordering is well resolved only from 25% upwards; the conclusions above accordingly rest on the trend across all five sizes rather than on any individual pair of entries. These bounds were established on a single specimen with one defect location and one set of machined geometries and characterise the present dataset rather than serving as general thresholds. Because the subsampled pools are drawn from the same acquisition sessions as the full pool, increasing the fraction adds correlated recordings rather than independent realisations, so the curve describes the effect of recording count and not that of independent experimental information.

#### 3.2.7. Confusion Analysis

**Figure 6:**
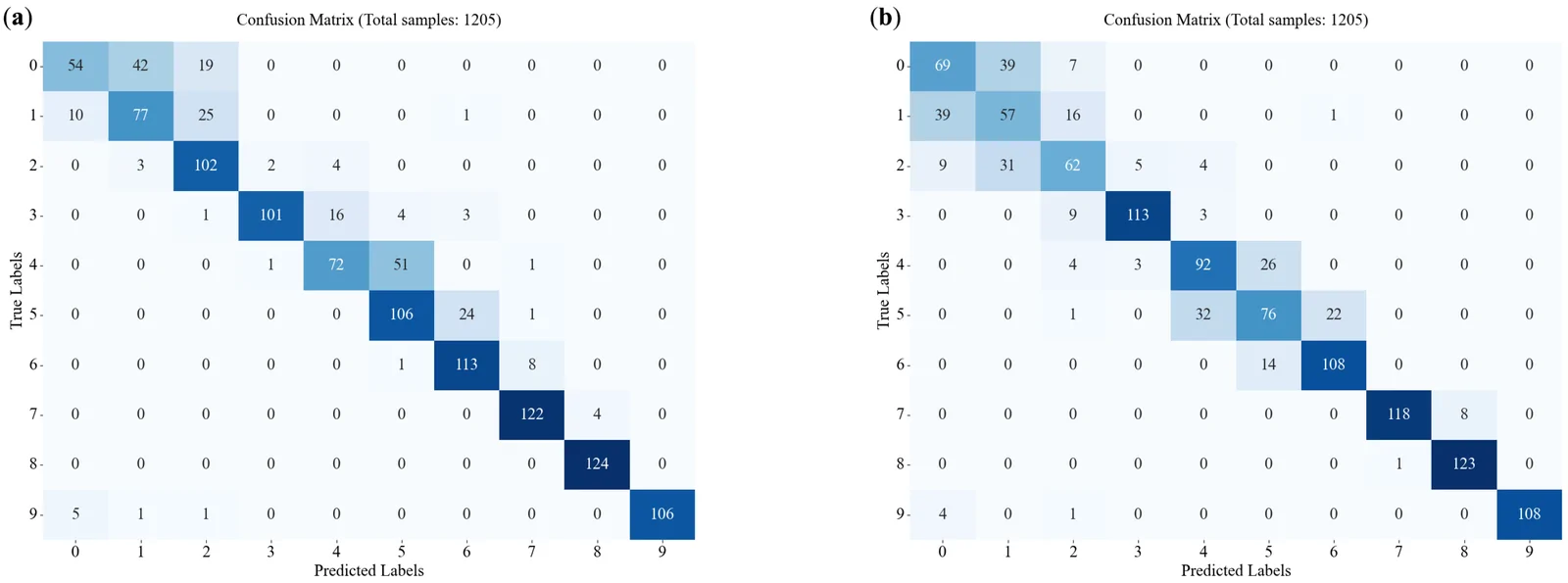
Row-normalised confusion matrices (i.e., per-class recall) on the test set under white noise at SNR = 0 dB for a representative run (seed 0). The colour scale encodes the per-class recall, with darker cells corresponding to higher values; the colour bar is shown to the right of each panel. Class labels follow Table 2: 0, Undamaged; 1–4, Abrasion 1–4; 5–8, Hole 1–4; 9, Hole 4 with an additional crack. (**a**) Mamba-2; (**b**) MCA-Mamba-2.

Figure 6 displays the performance comparison between Mamba-2 and the proposed technique under 0 dB white-noise conditions. The proposed method yields 81.09% test accuracy (80.95% macro-averaged recall), which represents 4.24 percentage points above Mamba-2 (4.42 points in terms of macro-averaged recall). Notably, the proposed method shows substantial improvements in recognising Abrasion 1 and Abrasion 2 (classes 1 and 2), whose recall increases by 17.7 and 36.0 percentage points respectively—from 50.4% to 68.1% and from 55.9% to 91.9%. Moreover, it markedly reduces the mutual confusion between these two lightly abraded states, which is severe under Mamba-2. Whereas Mamba-2 exhibits a noticeable advantage in class 4 (Abrasion 4, 73.6% versus 57.6%), the proposed method delivers superior overall performance and better classification balance across all categories.

### 3.3. Limitations

Several limitations should be borne in mind when interpreting the results. First, all measurements were obtained on a single 1.5 m steel pipe specimen with a 20 mm outer diameter, with defects machined at one fixed axial position (50 cm from the right end) and one fixed circumferential angle. It therefore cannot be determined from the present data whether the network learns generally transferable damage-related wave features or features specific to this arrangement, propagation distance and transducer coupling. Second, the transducers were re-coupled before the measurements of each damage state but not within a state, so exactly one coupling condition is associated with each class. Coupling variability is therefore present in the dataset but is confounded with the class label, and the present data cannot separate the contribution of the defect from that of the coupling condition under which it was measured. Third (and for the same reason), the dataset was partitioned at the level of individual records rather than by acquisition session: with a single session per damage state, a session-level partition would assign every sample of a class to one subset, and the task would degenerate. Strongly correlated records consequently appear in both the training and the test subset, and the reported accuracies are an upper bound in this respect as well. Both points would be resolved by acquiring several sessions per damage state with independent re-coupling, which would decouple the coupling condition from the class label and make a group-wise partition possible. Fourth, the noise was added synthetically according to prescribed spectral and distributional models; real measurement noise is non-stationary, may be correlated with the excitation, and generally comprises a mixture of contributions rather than a single analytical type. Fifth, in the matched-noise protocol, the training and test sets share the same noise type and intensity, so those results characterise classification performance at a given signal quality rather than robustness to unknown noise; the mismatched-noise experiments of Section 3.2.3 quantify the resulting gap. Sixth, no cross-specimen or in-service validation was performed. The performance reported here should therefore be regarded as an upper bound on what may be achieved under field conditions.

## 4. Conclusions

This study presented MCA-Mamba-2, a network that couples a convolutional front end with a bidirectional Mamba-2 state-space backbone and fuses features through combined multi-head self-attention and channel attention for the classification of ultrasonic guided wave signals acquired on a small-diameter steel pipe using a four-receiver array.

The following findings are directly supported by the experiments reported here. (i) On a ten-class dataset acquired on a 20 mm outer-diameter steel pipe specimen, MCA-Mamba-2 attained 98.01 ± 0.85% accuracy on noise-free signals and outperformed the RNN, LSTM, Transformer and Mamba-2 baselines at every noise type and SNR level tested, with the largest margin over Mamba-2 being 17.87 percentage points under pink noise at 5 dB. Because the dataset was partitioned at the level of individual records within a single acquisition session per damage state, these accuracies constitute an upper bound on the performance attainable on independently acquired data and should not be interpreted in isolation from this limitation. (ii) The ablation study attributes the improvement principally to the convolutional mixing block, which contributes 2.18 percentage points on noise-free signals, whereas multi-head self-attention contributes 1.62 percentage points; bidirectional state-space processing accounts for a further 0.76 percentage points, and the channel-attention branch accounts for 1.27 percentage points. (iii) Perhaps the most practically significant finding is the cross-noise evaluation: under the mismatched-noise protocol, accuracy decreases by 59.80 percentage points, on average, at 0 dB when the training and testing noise types differ, demonstrating that the high matched-noise accuracies do not transfer to unseen noise conditions and that noise-type mismatch, rather than noise intensity alone, is the dominant factor limiting deployment performance. Training a single model on a mixture of all four noise types and all four SNR levels effectively mitigates this gap, raising the average over the sixteen test conditions from 51.11% to 91.21%, and is therefore recommended as the default training protocol.

The following statements remain working hypotheses that the present data cannot confirm. The architecture is expected to transfer to other pipe diameters, wall thicknesses and defect positions, but this was not tested. Likewise, whether the network learns generally applicable damage-related wave features or features specific to the present experimental arrangement, propagation distance and transducer coupling cannot be resolved without cross-specimen measurements. The comparative advantage of state-space architectures over recurrent and attention-based ones is established here only for this benchmark and should not be read as a general claim about one-dimensional time-series processing.

Future work will address the limitations set out in Section 3.3 through measurements on multiple specimens and defect positions; through repeated acquisition sessions with independent transducer re-coupling for each damage state, which would decouple the coupling condition from the class label and permit a session-level partition; through the acquisition of in situ noise to replace the synthetic noise model; and through extension of the method to two-dimensional time–frequency representations for multi-modal fusion.

## Figures and Tables

**Table 1:** Layer-by-layer configuration of MCA-Mamba-2. *B* denotes the batch size, and *L* is the input sequence length.

Stage	Layer	Configuration	Output Shape
Input	–	Four receiver channels—R1–R4	(B,4,L)
Stem	Conv1d	k=7, s=1, p=3, *c*: 4→64	(B,64,L)
	BatchNorm1d	–	(B,64,L)
	ReLU	–	(B,64,L)
MambaConvBlock 1	Conv1d + BN + ReLU	k=3, *c*: 64→64	(B,64,L)
	BiMamba-2	$d_{\mathrm{state}}=8$, chunk = 16, $d_{\mathrm{model}}=128$, layers = 1	(B,128,L)
	Conv1d + BN + ReLU	k=3, *c*: 128→128	(B,128,L)
	Dropout	p=0.1	(B,128,L)
	Residual	1×1 conv, 64→128	(B,128,L)
	MaxPool1d	k=2, s=2	(B,128,L/2)
MambaConvBlock 2	Conv1d + BN + ReLU	k=3, *c*: 128→128	(B,128,L/2)
	BiMamba-2	$d_{\mathrm{state}}=8$, chunk = 16, $d_{\mathrm{model}}=128$, layers = 1	(B,128,L/2)
	Conv1d + BN + ReLU	k=3, *c*: 128→256	(B,256,L/2)
	Dropout	p=0.1	(B,256,L/2)
	Residual	1×1 conv, 128→256	(B,256,L/2)
	MaxPool1d	k=2, s=2	(B,256,L/4)
AttentionPoolingBlock	Multi-head self-attn	heads =⌊256/64⌋=4, dropout =0.1	(B,256,L/4)
	Channel attention	SE style, 256→64→256, sigmoid gate	(B,256,L/4)
	Conv1d + BN + ReLU	k=1, *c*: 256→128	(B,128,L/4)
	Dropout	p=0.1	(B,128,L/4)
Classifier	Conv1d + BN + ReLU	k=3, *c*: 128→256	(B,256,L/4)
	AdaptiveAvgPool1d	Output size of 1	(B,256)
	Linear + ReLU	256→128	(B,128)
	Dropout	p=0.5	(B,128)
	Linear	128→10	(B,10)
Total trainable parameters			1,182,586

**Table 2:** The dataset acquired in the experiment. The wall thickness of the specimen is 3.0 mm, and its cross-sectional area is 160.2 mm^2^. Abrasions are non-through-wall notches leaving a 1.0 mm ligament, whereas the 3.5 mm deep holes penetrate the wall completely. The area loss is evaluated at the axial plane through the defect centre.

Class	Depth	Width	Ligament	Through-Wall	Area Loss	Amounts
0 Undamaged	–	–	3.0 mm	No	0%	1130
1 Abrasion 1	2 mm	3 mm	1.0 mm	No	3.74%	1151
2 Abrasion 2	2 mm	5 mm	1.0 mm	No	6.24%	1126
3 Abrasion 3	2 mm	7 mm	1.0 mm	No	8.74%	1112
4 Abrasion 4	2 mm	9 mm	1.0 mm	No	11.23%	1246
5 Hole 1	3.5 mm	3 mm	0	Yes	5.62%	1253
6 Hole 2	3.5 mm	5 mm	0	Yes	9.36%	1309
7 Hole 3	3.5 mm	7 mm	0	Yes	13.11%	1220
8 Hole 4	3.5 mm	9 mm	0	Yes	16.85%	1258
9 Hole 4 add crack	3.5 & 1 mm	9 & 3 mm	0/2.0 mm	Yes/No	16.85% + 1.87%	1242
Total						12,047

**Table 3:** Definition of the four synthetic noise types along the two independent axes of power spectral density and amplitude distribution.

Noise Type	Power Spectral Density	Amplitude Distribution
White	Flat	Uniform (non-Gaussian)
Pink	∝1/|f|	Gaussian
Gaussian	$\propto 1/{\left|f\right|}^{2}$	Gaussian
Laplacian	Flat	Laplace (non-Gaussian, heavy-tailed)

**Table 4:** Classification accuracy of different models on noise-free data. Values are mean ± standard deviation over three independent runs with seeds 0–2. The best value in each column is shown in bold.

Method	Accuracy (%)	Macro-F1 (%)	Parameters
RNN	80.35 ± 5.98	79.62 ± 6.15	23,338
LSTM	88.72 ± 4.25	88.05 ± 4.40	75,978
Transformer	87.56 ± 2.31	86.88 ± 2.55	52,522
Mamba-2	92.15 ± 1.62	91.48 ± 1.80	127,910
Mamba-2 (width-matched)	95.30 ± 1.05	94.85 ± 1.25	1,123,902
MCA-Mamba-2	**98.01** ± 0.85	**97.65** ± 1.02	1,182,586

**Table 5:** Identification accuracy of each model on different noisy datasets. All entries are test accuracy (%), mean ± standard deviation over three independent runs (seeds 0–2). The best value of each column within a group is presented in bold.

Noise Type	Model	0 dB	5 dB	10 dB	15 dB
White	RNN	56.35 ± 4.16	64.56 ± 7.85	72.95 ± 6.35	78.92 ± 6.66
	LSTM	66.31 ± 2.42	79.42 ± 2.63	86.47 ± 1.88	87.39 ± 4.70
	Transformer	69.71 ± 2.82	82.66 ± 3.92	86.89 ± 3.39	86.22 ± 2.59
	Mamba-2	76.85 ± 2.95	77.93 ± 2.60	85.98 ± 1.70	89.79 ± 1.91
	MCA-Mamba-2	**81.09** ± 1.74	**86.14** ± 1.29	**90.95** ± 1.03	**96.02** ± 1.99
Pink	RNN	19.92 ± 4.36	59.67 ± 5.11	71.12 ± 7.62	72.03 ± 8.91
	LSTM	51.95 ± 2.17	64.73 ± 4.65	77.84 ± 5.63	92.28 ± 5.61
	Transformer	58.42 ± 2.03	66.64 ± 2.89	77.18 ± 2.18	95.02 ± 2.45
	Mamba-2	70.29 ± 2.50	67.72 ± 1.28	88.46 ± 2.94	83.65 ± 2.45
	MCA-Mamba-2	**83.65** ± 1.34	**85.59** ± 1.76	**93.53** ± 1.28	**96.93** ± 1.85
Gaussian	RNN	43.65 ± 3.48	57.18 ± 6.77	74.77 ± 5.86	85.89 ± 5.55
	LSTM	65.73 ± 4.72	73.78 ± 4.19	86.56 ± 4.65	95.02 ± 4.03
	Transformer	68.38 ± 2.22	85.31 ± 2.98	88.63 ± 2.96	94.85 ± 3.41
	Mamba-2	77.18 ± 2.47	80.08 ± 1.13	90.29 ± 1.70	86.14 ± 1.78
	MCA-Mamba-2	**82.49** ± 0.20	**88.80** ± 1.13	**94.52** ± 1.80	**97.84** ± 1.54
Laplacian	RNN	28.13 ± 2.57	58.17 ± 4.84	72.78 ± 5.38	83.40 ± 4.55
	LSTM	65.39 ± 2.46	80.83 ± 2.74	85.15 ± 5.04	91.62 ± 5.87
	Transformer	67.55 ± 3.90	75.85 ± 4.06	84.15 ± 4.26	92.70 ± 1.70
	Mamba-2	74.94 ± 1.25	88.80 ± 2.37	93.61 ± 1.69	94.69 ± 2.21
	MCA-Mamba-2	**84.15** ± 1.04	**92.20** ± 1.77	**95.35** ± 2.08	**97.43** ± 0.22

**Table 6:** Generalisation of MCA-Mamba-2 to unseen noise conditions. Rows are grouped by the noise type used at test time; within each group, the five rows are the training conditions, the first four being single-noise models trained at 0 dB and the fifth being a single model trained on a random mixture of all four noise types and all four SNR levels. Entries are test accuracy (%), mean ± standard deviation over three independent runs (seeds 0–2); the best value of each column within a group is in bold, and the matched train/test entries are underlined.

Test Noise	Training Condition	0 dB	5 dB	10 dB	15 dB	Avg.
White	White (0 dB)	**85.71** ± 5.76	9.30 ± 0.00	9.30 ± 0.00	9.30 ± 0.00	28.41
	Pink (0 dB)	58.42 ± 11.86	25.75 ± 14.17	28.96 ± 23.04	29.04 ± 24.51	35.54
	Gaussian (0 dB)	9.08 ± 2.18	19.82 ± 8.58	36.54 ± 18.70	87.26 ± 12.78	38.18
	Laplacian (0 dB)	66.39 ± 22.94	9.30 ± 0.00	9.30 ± 0.00	9.30 ± 0.00	23.57
	Mixed noise	77.16 ± 2.97	**91.25** ± 4.48	**95.27** ± 5.05	**96.57** ± 5.52	**90.06**
Pink	White (0 dB)	13.10 ± 2.93	9.30 ± 0.00	9.30 ± 0.00	9.30 ± 0.00	10.25
	Pink (0 dB)	**92.58** ± 5.55	42.97 ± 16.68	30.01 ± 24.22	28.82 ± 25.35	48.59
	Gaussian (0 dB)	10.91 ± 1.05	45.85 ± 14.33	**95.16** ± 4.18	**96.93** ± 0.19	62.21
	Laplacian (0 dB)	12.40 ± 4.67	9.30 ± 0.00	9.30 ± 0.00	9.30 ± 0.00	10.08
	Mixed noise	83.22 ± 10.40	**92.28** ± 6.66	94.49 ± 6.04	95.99 ± 5.77	**91.49**
Gaussian	White (0 dB)	9.61 ± 0.46	9.30 ± 0.00	9.30 ± 0.00	9.30 ± 0.00	9.38
	Pink (0 dB)	82.97 ± 17.96	47.95 ± 24.08	30.56 ± 27.86	29.98 ± 27.33	47.87
	Gaussian (0 dB)	**97.84** ± 0.20	**97.50** ± 0.35	**98.10** ± 0.28	**98.05** ± 0.31	**97.87**
	Laplacian (0 dB)	11.49 ± 3.79	9.33 ± 0.05	9.30 ± 0.00	9.30 ± 0.00	9.86
	Mixed noise	90.42 ± 7.62	93.63 ± 5.74	94.66 ± 5.44	95.93 ± 5.35	93.66
Laplacian	White (0 dB)	49.47 ± 8.17	9.30 ± 0.00	9.30 ± 0.00	9.30 ± 0.00	19.35
	Pink (0 dB)	49.28 ± 16.03	27.96 ± 14.35	29.35 ± 22.86	28.82 ± 24.68	33.85
	Gaussian (0 dB)	8.83 ± 2.69	17.88 ± 8.41	35.96 ± 15.94	88.01 ± 11.42	37.67
	Laplacian (0 dB)	**90.37** ± 1.92	9.30 ± 0.00	9.30 ± 0.00	9.30 ± 0.00	29.57
	Mixed noise	75.94 ± 4.52	**91.20** ± 4.63	**94.88** ± 4.82	**96.48** ± 5.52	**89.62**

**Table 7:** Ablation study of MCA-Mamba-2. Test accuracy and macro-averaged F1 score (%) are reported as mean ± standard deviation over three independent runs with different random seeds. Components are added cumulatively from the A0 backbone along the chain of A0–A1–A2–A3–A5; A3 and A4 isolate the self-attention and channel-attention branches, respectively. The best result in each column is shown in bold.

Metric	Config	Noise-Free	White 0 dB	Pink 0 dB	Params
Accuracy	A0 (backbone)	92.18 ± 1.85	76.52 ± 1.33	76.80 ± 1.64	487,874
	A1 (+Conv)	94.36 ± 1.42	77.83 ± 6.06	78.45 ± 6.06	681,346
	A2 (+Bi-dir)	95.12 ± 1.08	79.15 ± 1.61	79.90 ± 1.77	886,330
	A3 (+MHSA)	96.74 ± 0.65	78.40 ± 5.24	81.20 ± 5.90	1,149,498
	A4 (+SE)	94.85 ± 2.30	70.28 ± 15.17	75.60 ± 0.75	919,418
	A5 (full model)	**98.01** ± 0.85	**81.09** ± 1.74	**83.65** ± 1.34	1,182,586
Macro-F1	A0 (backbone)	91.05 ± 2.10	75.20 ± 1.78	76.10 ± 1.44	487,874
	A1 (+Conv)	93.28 ± 1.65	76.50 ± 7.54	77.80 ± 6.49	681,346
	A2 (+Bi-dir)	94.16 ± 1.25	78.05 ± 2.01	78.95 ± 2.10	886,330
	A3 (+MHSA)	95.92 ± 0.78	77.30 ± 5.32	80.15 ± 6.32	1,149,498
	A4 (+SE)	93.74 ± 2.65	65.40 ± 19.46	74.20 ± 0.44	919,418
	A5 (full model)	**97.65** ± 1.02	**79.85** ± 1.90	**82.40** ± 1.50	1,182,586

A0: backbone with residual connections only; A1: A0 + convolutional mixing block; A2: A1 + bidirectional Mamba-2; A3: A2 + multi-head self-attention; A4: A2 + channel (squeeze-and-excitation) attention; A5: full model with both attention branches. All values are averaged over three seeds. The A5 entries at 0 dB coincide with the corresponding MCA-Mamba-2 entries of Table 5.

**Table 8:** Hyperparameter sensitivity and convergence analysis. The learning-rate sweep fixes batch size = 4 (effective = 16); the batch-size sweep fixes $\mathrm{lr}=10^{-3}$. All runs use 40 epochs with seed = 0. “Best at 20” denotes the highest test accuracy reached within the first 20 epochs.

		Clean		0 dB White Noise		
Sweep	Configuration	Acc. (%)	Best at 20 (%)	Acc. (%)	Best at 20 (%)	Best Epoch
Learning rate	$\mathrm{lr}=1\times 10^{-4}$	96.42	96.42	76.85	76.85	5/18
	$\mathrm{lr}=5\times 10^{-4}$	97.18	97.18	79.42	79.20	3/19
	$\mathrm{lr}=1\times 10^{-3}$ ^†^	**98.01**	**98.01**	**81.09**	**81.09**	9/20
	$\mathrm{lr}=5\times 10^{-3}$	82.47	82.10	41.83	40.55	13/28
Batch size	bs=4 (eff. 16) ^†^	**98.01**	**98.01**	81.09	81.09	9/20
	bs=16 (eff. 64)	97.65	97.60	**83.65**	**83.65**	21/28

Bold indicates the best value in each column. ^†^ Default configuration used in this paper. Best epoch is reported as clean/noisy.

**Table 9:** Effect of the training-set size on classification performance (%) under white noise at 0 dB. The training pool is subsampled class-wise to the indicated fraction, while the test set is kept complete; the ten-class label set is unchanged, and the subsets are nested. Entries are mean ± standard deviation over three independent runs (seeds 0–2), and Δ denotes the difference of the means, i.e., MCA-Mamba-2 minus Mamba-2.

Training Pool	Fraction	Accuracy			Macro-F1		
		Mamba-2	MCA-Mamba-2	Δ	Mamba-2	MCA-Mamba-2	Δ
482	5%	64.87 ± 4.82	**68.36** ± 3.15	+3.49	59.16 ± 5.40	**62.30** ± 4.28	+3.14
964	10%	70.02 ± 3.96	**73.26** ± 2.74	+3.24	65.92 ± 4.35	**69.59** ± 3.62	+3.67
2409	25%	74.58 ± 3.21	**77.85** ± 2.28	+3.27	71.20 ± 3.65	**74.90** ± 2.95	+3.70
4819	50%	75.62 ± 3.05	**79.33** ± 1.96	+3.71	72.55 ± 3.42	**76.80** ± 2.50	+4.25
9637	100%	76.85 ± 2.95	**81.09** ± 1.74	+4.24	73.90 ± 3.20	**79.85** ± 1.90	+5.95

Bold indicates the better of the two models in each column. All entries are means over three independent runs with seeds 0–2, following the protocol used throughout the paper. The 100% row consequently reproduces the values already reported for this condition: its accuracies in the white-noise 0 dB column of Table 5 and its macro-F1 for MCA-Mamba-2 in configuration A5 of Table 7.

## Data Availability

The data presented in this study are available on request from the corresponding author.

## Abbreviations

The following abbreviations are used in this manuscript:

UGW	Ultrasonic Guided Wave
SHM	Structural Health Monitoring
NDT	Non-Destructive Testing
FEM	Finite Element Method
CNN	Convolutional Neural Network
RNN	Recurrent Neural Network
LSTM	Long Short-Term Memory
SNR	Signal-to-Noise Ratio
PSD	Power Spectral Density
SSD	Structured State-Space Duality
MHSA	Multi-Head Self-Attention
SE	Squeeze and Excitation
VAE	Variational Auto-Encoder
MCA	Multi-Channel Attention
LIME	Local Interpretable Model-Agnostic Explanations
